# Chemokines as the modulators of endometrial epithelial cells remodelling

**DOI:** 10.1038/s41598-019-49502-5

**Published:** 2019-09-10

**Authors:** A Złotkowska, A Andronowska

**Affiliations:** Department of Hormonal Action Mechanisms, Institute of Animal Reproduction and Food Research of the Polish Academy of Sciences, Olsztyn, Poland

**Keywords:** Cell adhesion, Cell migration, Cellular imaging

## Abstract

Previous studies highlighted chemokines as potential factors regulating changes in the endometrium during early pregnancy. The current study aimed to screen the effects of a broad range of chemokines and indicate those that are involved in porcine luminal epithelial (LE) cell remodelling. Messenger RNA expression of chemokines (*CCL2*, *CCL4*, *CCL5*, *CCL8*, *CXCL2*, *CXCL8*, *CXCL10* and *CXCL12)* and both the mRNA and protein expression of their receptors (CCR1, CCR2, CCR3, CCR5, CXCR2, CXCR3, CXCR4) were detected in LE cells. Exogenous CCL8 enhanced the proliferative and migration potential of LE cells and their motility in the environment with its stable concentration. The adhesive properties of LE cells were negatively affected by CCL8. However, CXCL12 positively affected the proliferation, motility and adhesion of LE cells as well as caused a decrease in MUC1 mRNA expression. To conclude, our studies determined that exogenous chemokines affected critical endometrial epithelial cell functions in the context of embryo implantation. We suggest that of all the examined factors, chemokine CCL8 participates in the establishment of a proper environment for embryo implantation, whereas CXCL12, apart from participation in endometrial receptivity, promotes embryo attachment.

## Introduction

Pregnancy proceeds differently depending on the species. In pigs, the peri-implantation period, when embryo mortality is the highest (approximately 30%), decides the success of a pregnancy^1^. Enhanced foetal loss during the mentioned period happens when there is inappropriate embryo development or disrupted communication between mother and embryo, which can be the effect of inadequate endometrial preparation for embryo attachment^2^.

The endometrium responds to trophoblast signals only after reaching endometrial receptivity. The main factors that control this process are steroid hormones produced by ovaries. Endometrial receptivity is associated with decreased expression of mucin 1 (MUC1) on the surface of luminal epithelial cells and enhanced expression of adhesive molecules such as integrins^3^. Additionally, osteopontin (SPP1), which is intensively produced by luminal epithelial (LE) cells, acts in both a paracrine and autocrine manner on trophoblast and epithelial cells, respectively, facilitating proper communication^4–6^_._ In addition to the mentioned alterations that allow embryo attachment, the luminal epithelium structure also changes^7^. Many factors such as prostaglandins, growth factors and various cytokines/chemokines may modulate endometrial functions and play a role in the preparation for appropriate embryo implantation^8^.

Chemokines are proteins with the ability to control mainly immune cell chemoattraction; however, they may also participate in cell proliferation, migration and apoptosis^9,10^. Based on their structure, which is associated with the localization of two cysteine residues in the N-terminus, chemokines are divided into the groups XC, CC, CXC and CX3C and possess the ability to bind and transduce signals through G protein-coupled receptors^11^. Chemokines are believed to play important roles at the porcine maternal-foetal interface^12,13^; however, their function in endometrial rearrangement and maternal-embryo communication is still poorly understood. As there are few studies concerning their involvement during pregnancy in species with invasive placentation^14,15^, it is possible that in species with non-invasive placentation, such as pigs, chemokines also may highly contribute to appropriate endometrial remodelling and trophoblast development.

The aim of this study was to evaluate a broad range of chemokines (CCL2, CCL4, CCL5, CCL8, CXCL2, CXCL8, CXCL9, CXCL10, CXCL12) and indicate those that are involved in porcine luminal epithelium remodelling. We hypothesize that chemokines control LE cell proliferation, migration and adhesion and subsequently positively or negatively regulate embryo implantation.

## Material and Methods

### Epithelial cells isolation

Porcine uteri from the mid-luteal phase (days 8–12) of the oestrous cycle were collected from the local abattoir. The approximate day of the oestrous cycle was determined based on the corpus luteum colour and morphology^16^. Pieces of endometrial tissue from both horns of the uterus were washed in phosphate-buffered saline (PBS; NaCl, KCl, Na_2_HPO_4_, KH_2_PO_4_) and digested in 0.2% dispase (in Dulbecco’s PBS) (Sigma Aldrich, Germany) for 50 min at 37 °C with continuous stirring. The obtained cell suspension was filtered through 270-µm mesh to separate the remaining fragments of the tissue. Cells were suspended in M199 medium supplemented with penicillin/streptavidin (P/S) and 5% normal calf serum (NCS) (Sigma Aldrich, Germany), and the centrifugation procedure was repeated three times (10 min, 1100 rpm). Then, the cell suspension was filtered through a 100-µm cell strainer (Becton Dickinson, USA), and the fraction that passed through it was collected. The obtained cells were seeded in 75-cm^3^ culture flasks (2 million cells per 1 ml of medium) and incubated for 5 h (37 °C/5% CO_2_) in M199 medium with P/S and 10% NCS. After this period, non-attached epithelial cells were collected and seeded on new collagen-coated culture dishes. Cell purity was checked by immunofluorescence staining for cytokeratin (C9687, Sigma Aldrich, Germany). The homogeneity of the cell population ranged from 90–100%.

### Proliferation analysis

Cells after first passage were seeded on 96-well collagen-coated plates (10.000 cells per well) in M199 with P/S and 10% NCS (n = 5). After 24 h and 70% confluence, attached cells were starved for 4 h in serum-free medium. Subsequently, cells were treated for 24 h with single mammalian recombinant chemokines (Peprotech, UK, Supplementary data 1) at a concentration of 1 ng/ml (diluted in PBS+ 0.1% BSA) suspended in M199 medium with P/S and 0.1% BSA (Sigma Aldrich, Germany). Negative controls without additional stimuli (M199+ 0.1% BSA+ adequate volume of PBS (diluent for chemokines)) as well as positive controls with NCS were included. Six technical repeats of each treatment were performed. The CellTiter 96® AQueous One Solution Cell Proliferation Assay was used to determine the ability of cells to proliferate (Promega, USA). The absorbance was measured at the wavelength 490 nm using a microplate reader. The results were analysed in comparison to the control group without additional stimuli.

### Migration analysis

Cells after the first passage were seeded (50.000 cells per insert) in serum-free medium on the upper side of 8-µm pore size culture inserts (24-well plate, Corning, USA) (n = 3). Medium in wells below the inserts was supplemented with single chemokines (1 ng/ml). Negative controls without additional stimuli as well as positive controls with NCS were included. After 24 h of incubation, cells attached to the basal side of the membrane were fixed in 4% formaldehyde and 100% ice-cold methanol. Then, cells from the apical side of the insert were removed using cotton sticks. Cells from the basal side were stained in 0.01% DAPI (Sigma Aldrich, Germany), and numbers of cells were counted automatically using ZEN 2.3 software under an Axio Observer inverted microscope (Zeiss, Germany). The results were analysed in comparison to the control group without additional stimuli.

### Adhesion analysis

Epithelial cells after the first passage were seeded in M199 medium supplemented with single chemokines (1 ng/ml) on a 96-well collagen-coated plate (10.000 cells per well) (n = 5, eight technical repeats of each treatment were performed). Plates were incubated for 5 h at 37 °C/5% CO_2_. Next, unattached cells were washed three times with pre-warmed PBS, and 0.2% crystal violet (in 10% EtOH) was added into each well for 5 min. Cells were washed again with PBS three times, and solubilizing buffer (NaH_2_PO_4_/50% EtOH) was added to each well to achieve total discoloration of cells. The absorbance was measured at a wavelength of 540 nm. Results were analysed in comparison to the control group without additional stimuli.

### Scratch assay

Epithelial cells after the first passage were seeded on collagen-coated 24-well plates in M199 with P/S and 10% NCS. After reaching full confluence, cells were starved for 4 h in serum-free medium. After this time, using 10-µl pipette tips, a 500-µm wide gap was manually created in the middle of each well. Wells were washed with pre-warmed PBS and fresh M199 medium with 0.1% BSA, and single chemokines (1 ng/ml) were added into each well. Each treatment was performed in duplicate (n = 5). Controls assigned as non-treated cells were also performed. An Axio Observer inverted microscope with ZEN 2.3 software was used to monitor the experiment (Zeiss, Germany). Pictures of whole gaps were taken every 2 hours in ‘tiles mode’. For exact analysis, pictures at 0, 6, 10, 18, and 24 h of the experiment were used. The parameter that was used for statistical analysis was calculated as the percent of gap closure in the subsequent hours of the experiment. The results were analysed in comparison to the control group without additional stimuli. An additional control group with proliferation stimulator (NCS) was included to distinguish the proliferative effect from the migratory response in the designed assay.

### Immunocytochemistry

Epithelial cells after the first passage were seeded in M199 with P/S and 10% NCS onto 8-chamber cell imaging coverglasses (Eppendorf, Germany) and cultured until reaching 70% confluence. Cells were washed three times with pre-warmed PBS and fixed in 4% paraformaldehyde (pH 7.45) for 20 min. Non-specific binding was blocked by 1 h of incubation in blocking buffer (0.1 M PBS, 0.1% BSA, 0.05% trimerosol) with 10% Normal Donkey Serum (Sigma Aldrich, Germany). Overnight incubation with primary antibodies was performed at 4 °C (Supplementary data 2). After washing in PBS, secondary antibodies were added into each well (donkey anti-rabbit antibodies conjugated to Alexa 488, Life Technologies, USA), and a 90-min incubation was performed in the dark. Then, washing in PBS was repeated three times, and cells were incubated in 0.01% DAPI (Sigma Aldrich, Germany) for 30 min. The plastic chamber was removed from each coverslip, which were mounted onto microscope slides (Menzel, Germany) using VECTASHIELD® Antifade Mounting Medium (Vector Laboratories, UK). Negative controls without primary, secondary and both antibodies as well as isotype controls were also included. Pictures were taken with an LSM 800 confocal microscope (63×/1.40 oil objective) (Zeiss, Germany).

### RNA isolation, reverse transcription and Real-Time PCR analysis

Isolation of RNA was performed with the commercial Total RNA Mini Plus Concentrator Kit (A&A Biotechnology, Poland) according to the manufacturer’s protocol. The quantity and purity of the isolated RNA were measured using a NanoDrop 100 (ThermoFisher Scientific Inc, DE, USA), whereas RNA quality was assessed using a Bioanalyzer Agilent 2100 (Agilent Technologies, Germany). RNA was stored at −80 °C for further use. Reverse transcription was performed using the High Capacity cDNA Reverse Transcription Kit (ThermoFisher Scientific, USA), according to the manufacturer’s protocol. Real-Time PCR was performed using a Viia7 Real-Time PCR System (Applied Biosystems, USA) on 384-well plates (ThermoFisher Scientific, USA). Expression of chemokines and receptors was determined using TaqMan with the UNG assay and specific probes (Supplementary data 3), whereas expression of other genes (*MUC1*, *SPP1*, *TJP1*) was measured using the SybrGreen system with designed primers (Supplementary data 4). Identity of the amplified product was confirmed by sequencing (Genomed, Poland). All samples were run in duplicate. For each examined gene, three controls were assessed: two controls for reverse transcription (one with nuclease-free water instead of template and with reverse transcriptase and the second without reverse transcriptase) and a control with nuclease-free water instead of template. Relative quantification was performed, and the double delta Ct method was used for calculation. Data were normalized to two reference genes, ACTB and GAPDH.

### Statistical analysis

Statistical analysis was performed using GraphPad Prism 7 (GraphPad Software, Inc., USA). A normal distribution was verified using the Shapiro-Wilk test. Analysis of proliferation, migration and adhesion as well as gene expression analysis were performed using one-way ANOVA, followed by the Least Significant Difference post hoc test. The results from the scratch assay were analysed using two-way analysis of variance with repeated measures followed by the LSD post hoc test. The significance level (alpha) of each test was set to 0.05, and differences were considered significant if p < 0.05.

### Conference presentation

Presented in part at the “*In vitro* 3-D Total Guidance and Fitness” Proceedings of the CellFit workshop, 09–12 April 2018, Tartu, Estonia.

## Results

### Chemokines and the expression and localization of their receptors in luminal epithelial cells

**Figure 1:**
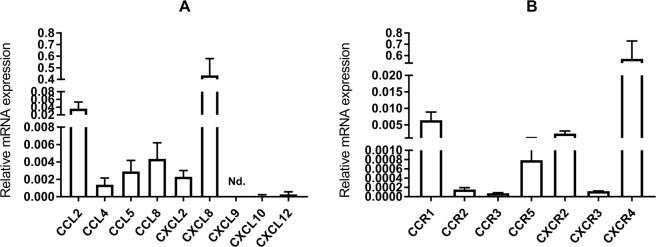
Relative mRNA expression of chemokines (**A**) and their receptors (B) in primary epithelial cells after first passage (n = 5). Nd.- expression not detected. Data are expressed as the mean ± SEM.

**Figure 2:**
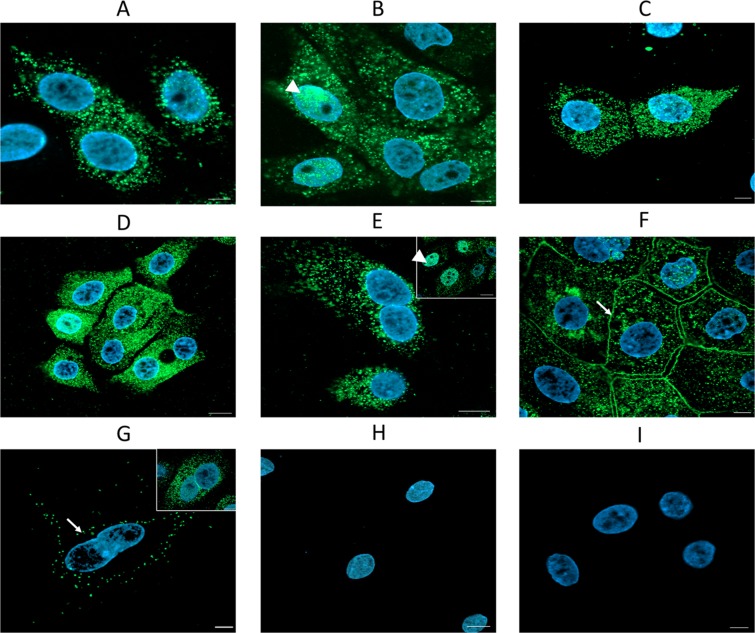
Immunolocalization of chemokine receptors in primary endometrial epithelial cells (A-CCR1, B-CCR2, C-CCR3, D-CCR5, E-CXCR2, F-CXCR3, G-CXCR4, H-control without primary antibody, I-isotype control). Arrow, cell membrane; arrowhead, nuclei. Scale bar: 20 µm.

Gene expression of all examined chemokines (CCL2, CCL4, CCL5, CCL8, CXCL2, CXCL8, CXCL10, CXCL12) was detected in isolated primary LE cells after the first passage except for *CXCL9*, for which its gene expression was under the detection limit (Fig. 1A). To assess the influence of chemokines on endometrial LE cells, it was necessary to confirm the presence of their receptors in those cells. Gene expression analysis revealed the presence of all examined receptors (CCR1, CCR2, CCR3, CCR5, CXCR2, CXCR3 and CXCR4) in LE cells (Fig. 1B). Immunofluorescence staining allowed all receptors to be localized in primary LE cells cultured *in vitro*. The presence of receptors CCR1, CCR3 and CCR5 was observed mainly in the cell cytoplasm (Fig. 2A,C,D), whereas receptors CCR2 and CXCR2 were found in the cytoplasm of all cells and in the nuclei of some cells (Fig. 2B,E). Receptor CXCR3 was localized in the membranes and cytoplasm of all cells (Fig. 2F). Some single cells had CXCR4 expression only in the cell membrane, whereas other cells had expression in the cytoplasm (Fig. 2G). The confirmation of chemokine receptor expression at both the mRNA and protein levels in LE cells allowed experiments to be conducted further, in which cells were treated with ligands for those receptors.

### Proliferation, migration and adhesion of luminal epithelial cells

**Figure 3:**
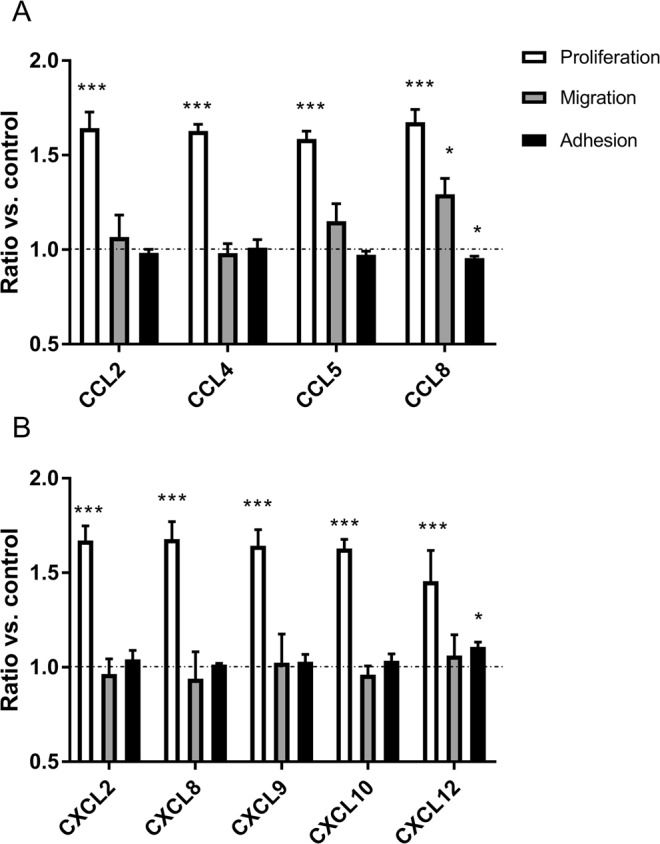
Effect of treatment with –CC– (**A**) and –CXC– (**B**) chemokines on proliferation (n = 5), migration (n = 3) and adhesion (n = 5) of luminal epithelial cells. Statistical analysis was performed as comparison of results after stimulation with single chemokine to control group without any stimuli (signed as horizontal line). Asterisks indicate a significant difference in comparison to control group (*p < 0.05, ***p < 0.001). All data are expressed as the mean ± SEM. Results are presented on the same graph only to enable better analysis, but statistical analysis was performed separately for each assay (proliferation, migration, adhesion).

As the presence of receptors was confirmed in LE cells, experiments with their ligands were conducted. To determine the effect of chemokines on LE cell behaviour, their physiological concentration (1 ng/ml) was used. All examined chemokines significantly enhanced LE cell proliferation compared with that of non-treated cells assigned to the control group (p < 0.001). The transwell migration assay revealed that the CCL8 gradient created across the membrane caused enhanced LE cell migration towards the higher chemokine concentration (p < 0.05). The adhesive properties of LE cells were affected by CCL8, which significantly decreased their attachment to a collagen-coated surface, whereas CXCL12 enhanced the number of cells attached to the surface (p < 0.05) (Fig. 3A,B).

### Motility of luminal epithelial cells in a scratch assay

**Figure 4:**
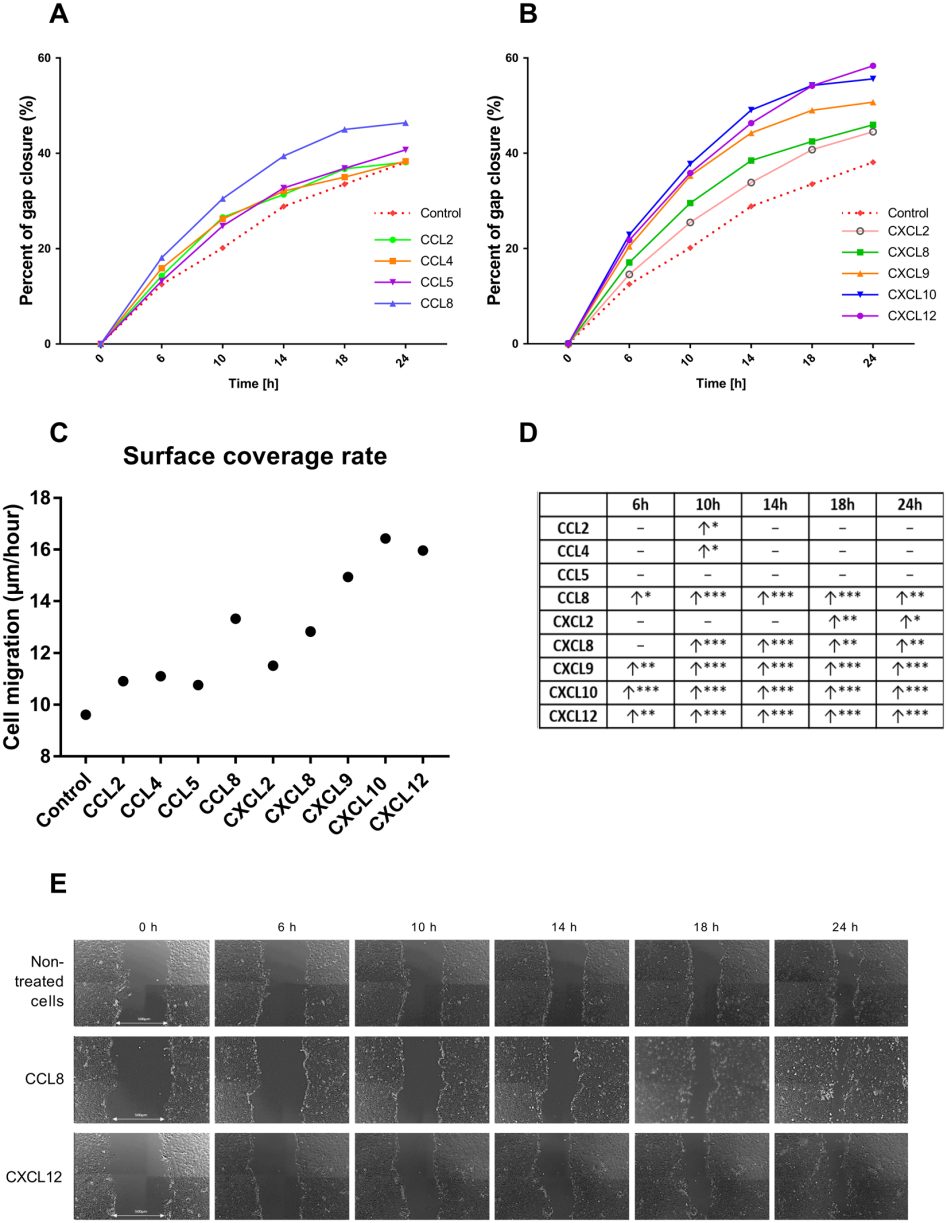
Analysis of primary epithelial cells motility in scratch assay after stimulation with –CC– (**A**) and –CXC– (**B**) chemokines (n = 5). All data are expressed as the mean for better clarity of the graph. Surface coverage rate was calculated as the area covered by cells in time unit (µm/hours) (**C**). All data are presented in table (**D**). Arrow pointing up indicate significantly increased closure of the gap area after chemokine treatment in compared with non-treated cells (red dotted line) in specific time points (6 h, 10 h, 14 h, 18 h, 24 h) (*p < 0.05, **p < 0.01, ***p < 0.001). Horizontal line indicate lack of statistically significant difference between treated and non-treated cells. Pictures representing gap closure area for non-treated, CCL8- and CXCL12- treated LE cells in different time points (**E**).

To assess the nature of LE cells in an environment with stable chemokine concentrations, a scratch assay was conducted. To confirm if the scratch assay determined mostly cell migration or proliferation, a control with a stimulator of epithelial cell proliferation (NCS) was performed (Supplementary data 5). The gap closure area of NCS-treated cells did not differ significantly from the area of non-treated cells considered the negative control (p > 0.05), indicating that most cells counted in the area of the gap had migrated there from the regions adjacent to the gap. Thus, the results are considered indicative of cell migratory potential in an environment with a stable chemokine concentration. Consequently, the concentration of 1 ng/ml was used for all chemokines in this experiment. LE cells stimulated with CCL2 and CCL4 showed a slightly increased migration ability in the tenth hour of incubation compared with that of the control group without additional stimuli (p < 0.05). Chemokines CCL8, CXCL9, CXCL10 and CXCL12 had a strong positive effect on LE cell motility at all examined time points. CXCL2 increased cell movement in the 18^th^ and 24^th^ hours, whereas CXCL8 stimulated cell motility from the 10^th^ to 24^th^ hour of incubation (Fig. 4). The only chemokine that did not influence the potential of epithelial cells for gap closure was CCL5 (p > 0.05).

### Genes associated with endometrial receptivity

**Figure 5:**
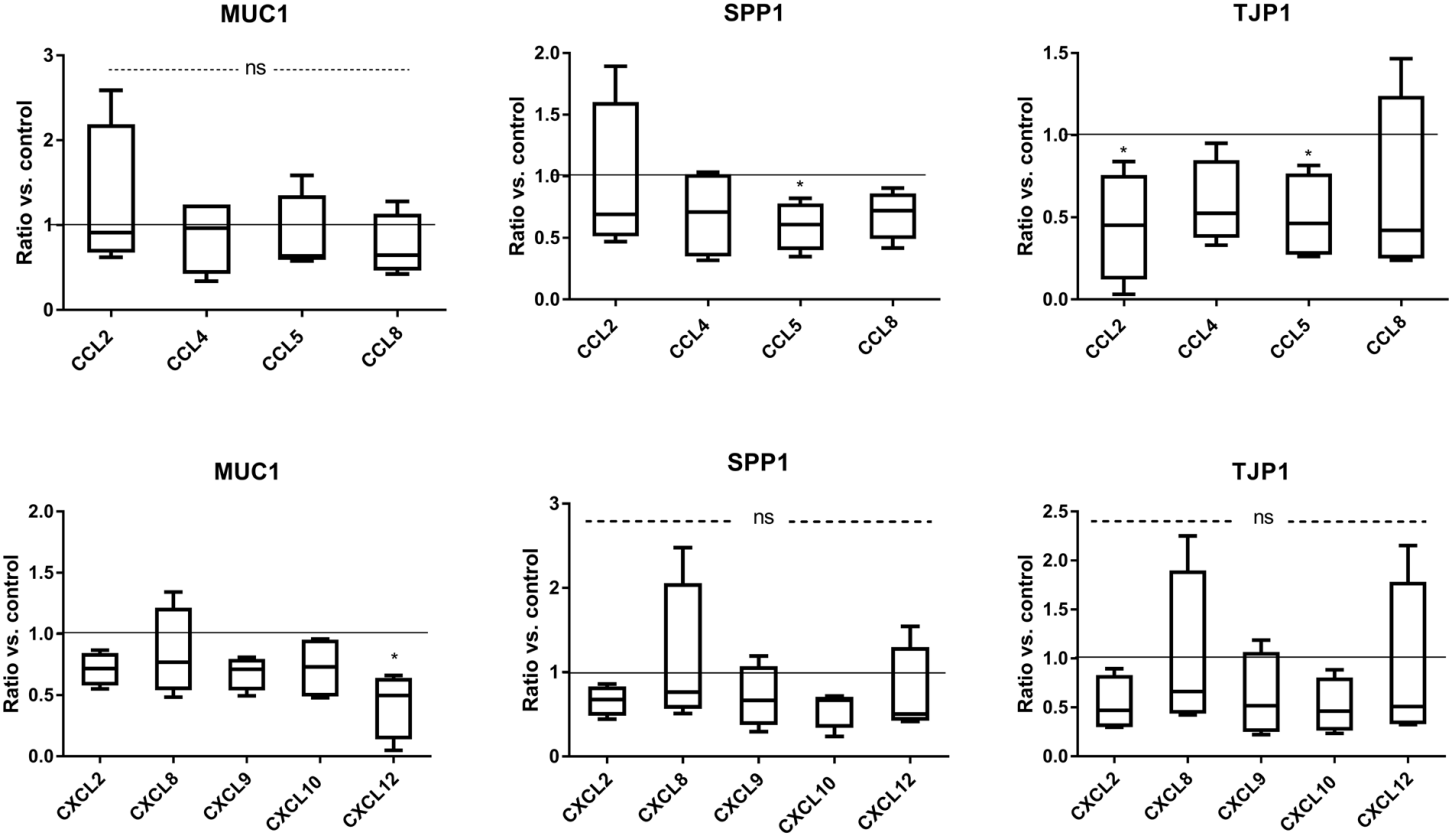
Effect of chemokine stimulation on *MUC1*, *SPP1* and *TJP1* gene expression in luminal epithelial cells (n = 5). Asterisks indicate statistically significant difference in comparison to control group (horizontal line) (*p < 0.05). Ns- non significant. All data are expressed as the mean with 5–95 percentile.

To evaluate the role of chemokines in enhancing endometrial receptivity, real-time PCR analysis was performed, and the expression of several genes participating in this process was determined. Regarding the effect of LE cell stimulation with individual chemokines, CCL5 was revealed to downregulate *SPP1* and *TJP1* mRNA expression in epithelial cells, whereas CCL2 decreased *TJP1* expression relative to non-treated cells (p < 0.05). *MUC1 mRNA* expression significantly decreased after CXCL12 stimulation (p < 0.05). The other examined chemokines did not affect *MUC1*, *SPP1* and *TJP1* gene expression (p > 0.05) (Fig. 5).

## Discussion

Endometrial remodelling occurs cyclically during the oestrous cycle and during pregnancy and coincides with changes in the luminal epithelium, stroma and endothelium of blood vessels. Chemokines likely participate in the mentioned changes. Their physiological and pathological content in human blood serum in most cases varies between 0.1–1 ng/ml^17^, whereas the CXCL12 concentration in porcine blood serum was approximately 1.467 ± 1.19 ng/ml (mean ± SD) (Supplementary data 6). To maintain chemokine concentrations close to their physiological values, all of them were used at a dose of 1 ng/ml in the current study. Here, we determined the expression of several chemokines and the distribution of their receptors in luminal epithelial cells. When the expression of endogenous chemokines was high, their specific receptors were observed to internalize to the cytoplasm and cell nuclei (CCR1,-2,-3-5 and CXCR2). Chemokines with low expression were unable to cause receptor internalization, and receptors remained in the cell membrane (CXCR3,-4). Ligand-induced internalization of G-protein coupled receptors is very common and may result from negative signalling regulation through the removal of the active receptor from the membrane or continued signal transmission, which is initiated at the plasma membrane^18^. Based on these findings, the next step aimed to determine the direct effects of several chemokines on LE cell proliferation, migration and adhesion and the expression of several genes that are important in embryo implantation and endometrial receptivity.

Chemokines are likely to take part in endometrial-trophoblast interactions and are responsible for appropriate implantation and/or rejection of the embryo^19^. The exact role of chemokines in porcine conceptus development and endometrial remodelling is poorly understood; however, differential expression with significantly increased gene expression in arrested conceptuses suggest their involvement in the control of embryo development^1,12^. Variable chemokine expression during the peri-implantation period was previously determined in porcine pregnant endometrium^13^. Because many chemokines, such as CCL2, CCL4, CCL5, CXCL9, and CXCL10, were found to be involved at the porcine maternal-foetal interface^13,20–22^, the current study was focused on screening the role of those chemokines in LE cells. Although several publications have dealt with the effect of those chemokines on trophoblasts of different species^14,21,23,24^, there is a deficit of studies that have determined their role in LE cells. The lack of receptors for chemokines CXCL2, CXCL8, CXCL9 and CXCL10 in porcine trophoblasts around the time of implantation eliminates them as potential factors facilitating trophoblast migration and development^13^. However, the presence of specific receptors for all of those chemokines was confirmed in porcine^13^ and bovine^25^ LE, not only during pregnancy but also during the oestrous cycle. At physiological concentrations, all chemokines caused an increase in LE cell proliferation, as indicated in the current study. Cyclic alterations in LE *in vivo* are connected with variations in cell-cell junctions. Non-permeable tight junctions between epithelial cells prevent the paracellular movement of molecules. Such prevention is important during the time of implantation^26^. An extensive branching network of strands is stabilized by proteins such as zonula occludens-1 (TJP1), which binds occludins and claudins with the actin cytoskeleton^27^. As indicated in this study, the expression of *TJP1* transcripts in LE cells was significantly decreased due to CCL2 and CCL5 stimulation, which suggests that these chemokines indirectly modify tight junctions between LE cells and make cell-cell connections more permeable. Thus, we suggest that the chemokines CCL2, CCL4, CCL5, CXCL2, CXCL8, CXCL9 and CXCL10, although they have the ability to act on LE cells, are not crucial factors that participate in intensive LE cell remodelling during porcine pregnancy. We suggest that their role in the behaviour of LE cells is equally divided between the oestrous cycle and pregnancy. However, there is a high probability that those chemokines during the peri-implantation period participate in other actions such as immune cell recruitment^22,28^, orangiogenesis^29^, but further studies are required to determine their exact role.

There has been no study concerning the role of CCL8 in porcine endometrial remodelling; however, we have previously demonstrated its action as one of the trophoblast-derived factors that is profusely released during maternal recognition of porcine pregnancy. Based on the determined expression and localization of specific receptors for CCL8 in endometrial tissue, its role in endometrial remodelling during early pregnancy is suggested and worth investigating^13^. The specific distribution and polarization of the CCR5 receptor in LE cells and its presence in human blastocysts implies the role of CCR5 ligands in embryo apposition and attachment^30^. However, the adhesive properties of porcine LE cells determined in the current study were negatively affected by exogenous CCL8. Thus, CCL8 may have had a slightly different effect on the implantation process in pigs than in other species. The proliferation and migration of endometrial LE cells in species with an invasive type of implantation are related to the creation of the space for embryonic penetration and reconstruction of the destroyed epithelial barrier^31^. In species with non-invasive implantation, such as pigs, all changes associated with the ability of epithelial cells to proliferate and migrate are probably focused on the preparation of an appropriate environment for embryo attachment and implantation. Here, we determined the positive effect of CCL8 on LE cell proliferation and migration towards stimuli, implying that twelve-day trophoblasts with elevated CCL8 expression may attract LE cells towards the area occupied by the trophoblasts and enhance epithelial folding. Moreover, the current study indicated that LE cells in an environment with a stable CCL8 concentration are more viable and strive to create an undisturbed layer, implying that CCL8 is involved in the preparation of LE for direct contact with the embryo. Taken together, chemokines that positively influence the creation of an appropriate implantation environment may negatively act on endometrial/embryo adhesive properties.

Both the endometrium and trophoblasts produce CXCL12, which may contribute to the establishment of pregnancy^13^. CXCL12-mediated effects on porcine trophoblast behaviour^32^ and human trophoblast invasion, survival and proliferation were previously characterized^33–36^. On the other hand, human first-trimester trophoblast cells were found to secrete high amounts of CXCL12 with the ability to bind to CXCR4 localized on decidual epithelial and stromal cells and positively influence the invasiveness and migration of decidual epithelial cells^37,38^. The paracrine effect of human CXCL12 produced by stromal cells on LE cells was previously described by Tsutsumi *et al*. (2011)^39^; however, thus far there have been no studies investigating the effect of CXCL12 on porcine LE cells. The upregulation of ligand and receptor was previously detected in the endometrium of species with non-invasive placentation such as sheep^40^ and pigs^13^ around the time of implantation. Our studies indicated that exogenous CXCL12 enhances LE cell proliferation and motility and subsequently leads to the creation of an undisturbed layer. As suggested by Wang *et al*. (2015), the CXCL12-CXCR4 axis is required for human blastocyst implantation and the progression of pregnancy^35^. Similarly, our findings revealed that porcine LE cell adhesion was positively affected after CXCL12 stimulation, which coincided with decreased *MUC1* expression in those cells. All these data imply that CXCL12 is the factor that participates in porcine endometrial preparation for embryo implantation, by controlling endometrial receptivity, and further embryo attachment.

In conclusion, our studies confirmed that chemokines are involved in LE cell remodelling. Among all of the examined factors, two play a more advanced role; CCL8 is suggested to participate in the establishment of a proper environment for embryo implantation, whereas CXCL12, apart from participation in enhancing endometrial receptivity, promotes embryo attachment.

## Supplementary information

Supplementary data

## Data Availability

The datasets generated during and/or analyzed during the current study are available from the corresponding author on reasonable request.
