# Author Correction: Chemokines as the modulators of endometrial epithelial cells remodelling

**DOI:** 10.1038/s41598-019-51009-y

**Published:** 2019-10-03

**Authors:** A. Złotkowska, A. Andronowska

**Affiliations:** Department of Hormonal Action Mechanisms, Institute of Animal Reproduction and Food Research of the Polish Academy of Sciences, Olsztyn, Poland

Correction to: *Scientific Reports* 10.1038/s41598-019-49502-5, published online 10 September 2019

The Acknowledgements section in this Article is incomplete.

“This study was supported by statutory activity of Institute of Animal Reproduction and Food Research of Polish Academy of Sciences in Olsztyn, Poland.”

should read:

“This study was supported by statutory activity of Institute of Animal Reproduction and Food Research of Polish Academy of Sciences in Olsztyn, Poland. The article processing charge was covered by the KNOW Consortium: “Healthy Animal - Safe Food” (Ministry of Sciences and Higher Education; Dec: 05-1/KNOW2/2015).”

