# Supplementary material for: Chemokines as the modulators of endometrial epithelial cells remodelling
Source: Sci Rep. 2019 Sep 10;9:12968. doi: 10.1038/s41598-019-49502-5 (PMC6736846; doi:10.1038/s41598-019-49502-5)
Supplement: Supplementary file 1 — Supplementary data [file 41598_2019_49502_MOESM1_ESM.pdf]

<u>Supplementary data</u>

# Title: Chemokines as the modulators of endometrial epithelial cells remodelling

**Złotkowska A, Andronowska A***

*Department of Hormonal Action Mechanisms, Institute of Animal Reproduction and Food Research of the Polish Academy of Sciences, Olsztyn, Poland*

***Corresponding author at**: Institute of Animal Reproduction and Food Research of Polish Academy of Sciences; Tuwima 10 Str., 10-748 Olsztyn, Tel. +48 89 523 46 86; fax +48 89 524. *E-mail: a.andronowska@pan.olsztyn.pl (Andronowska A)

# Supplementary Data 1: Recombinant chemokines used for in vitro studies

| Chemokine | Origin | Catalog number |
|---|---|---|
| **CCL2** | Human | 300-04 |
| **CCL4** | Human | 300-09 |
| **CCL5** | Human | 300-06 |
| **CCL8** | Murine | 250-14 |
| **CXCL2** | Murine | 250-15 |
| **CXCL8** | Human | 200-08 |
| **CXCL9** | Murine | 250-18 |
| **CXCL10** | Human | 300-12 |
| **CXCL12** | Murine | 250-20A |

# Supplementary Data 2: Antibodies used for immunocytochemistry

| Target protein | Dilution | Company | Catalog number |
|---|---|---|---|
| CCR1 | 1:50 | Santa Cruz Biotechnology | sc-7934 |
| CCR2 | 1:200 | Abcam | ab21667 |
| CCR3 | 1:50 | Santa Cruz Biotechnology | sc-7897 |
| CCR5 | 1:50 | Santa Cruz Biotechnology | sc-13950 |
| CXCR2 | 1:100 | Abcam | ab14935 |
| CXCR3 | 1:200 | Abcam | ab71864 |
| CXCR4 | 1:100 | Abcam | ab2074 |

# Supplementary Data 3: Probes used for real-time PCR

| Target gene | GeneBank accession number | Assay ID |
|---|---|---|
| **CCL2** | NM_214214.1 | Ss03394377_m1 |
| **CCL4** | NM_213779.1 | Ss03381395_u1 |
| **CCL5** | NM_001129946.1 | Ss03341385_u1 |
| **CCL8** | NM_001164515.1 | Ss04245586_m1 |
| **CXCL2** | NM_001001861.2 | Ss03378360_u1 |
| **CXCL8** | NM_213867.1 | Ss03392437_m1 |
| **CXCL9** | NM_001114289.2 | Ss03390033_m1 |
| **CXCL10** | NM_001008691.1 | Ss03391846_m1 |
| **CXCL12** | NM_001009580.1 | Ss03391855_m1 |
| **CCR1** | NM_001001621.1 | Ss03378192_u1 |
| **CCR2** | NM_001001619.1 | Ss03378154_u1 |
| **CCR3** | NM_001001620.1 | Ss03378176_u1 |
| **CCR5** | NM_001001618.1 | Ss03378121_u1 |
| **CXCR2** | AK237564.1 | Ss03376125_s1 |
| **CXCR3** | AJ851240.1 | Ss03375858_u1 |
| **CXCR4** | NM_213773.1 | Ss03392297_s1 |
| **B-actin** | AK237086.1 | Ss03376081_u1 |
| **GAPDH** | AF141959.1 | Ss03375435_u1 |

# Supplementary Data 4: Primers used for real-time PCR

| Target gene | Gene Bank accession number | Forward | Reverse | Melting temperature (ºC) |
|---|---|---|---|---|
| **MUC1** | XM_021089730.1 | CAGTGAAGTTAGTGCCTGGG | CCACAGTTCTTTCGTCGGC | 60 |
| **SPP1** | XM_005667005.3 | AAGAAGTTCCGCAGATCCGA | TGTCCAAGTCAGAAGCCACG | 60 |
| **TJP1** | XM_021098891.1 | CCCGAGAAGTTACGTGCTGAA | CGCTACGGCCCAGATAAACA | 60 |
| **B-actin** | U07786.1 | ACATCAAGGAGAAGCTCTGCTACG | GAGGGGCGATGATCTTGATCTTCA | 60 |
| **GAPDH** | KJ786424.1 | TCGGAGTGAACGGATTTG | CCTGGAAGATGGTGATGG | 60 |

**Supplementary Data 5: Control of epithelial cells proliferation with newborn calf serum (NCS) in the proliferation assay (A) and scratch assay (B).**

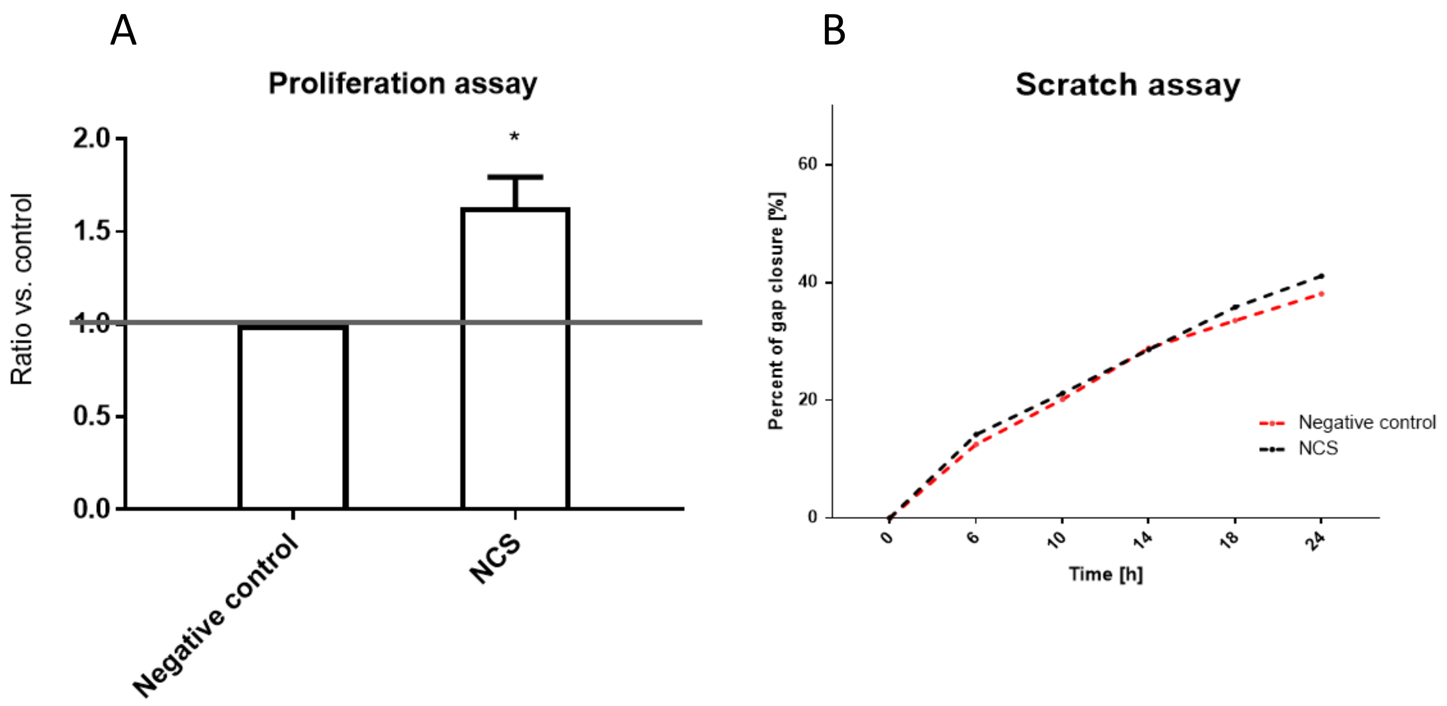

**Supplementary Data 6: Concentration of CXCL12 in porcine blood serum. All data are expressed as the mean±SD.**

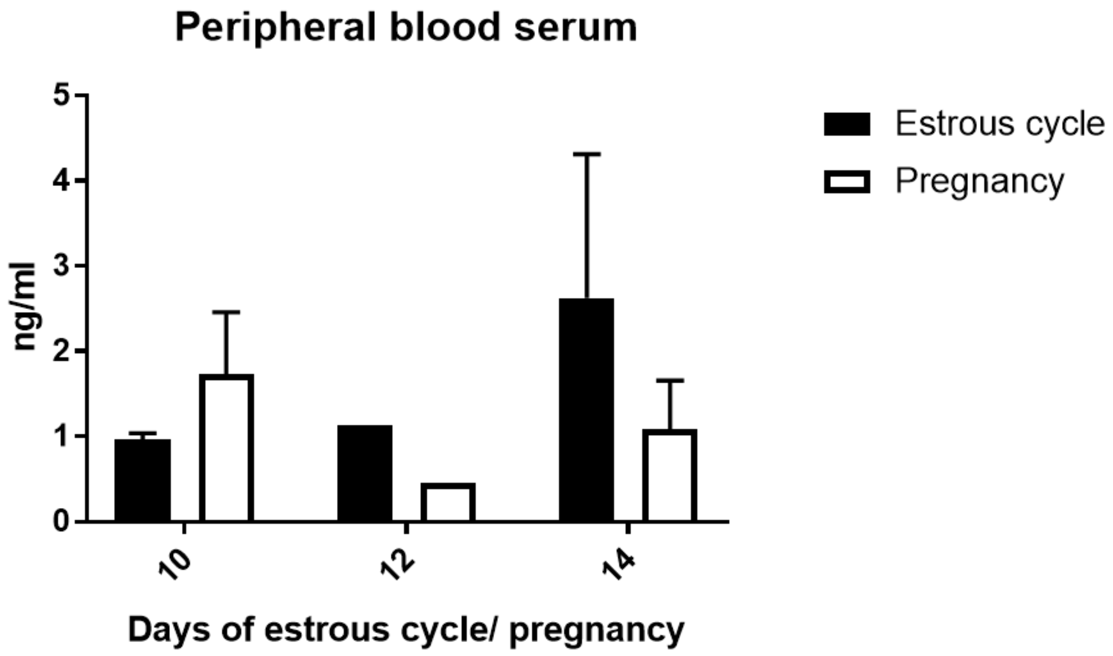


Mean±SD: 1,467±1,19 ng/ml
